# Case of Ovariotomy

**Published:** 1850-06

**Authors:** 


*Case of Ovariotomy.* By WASHINGTON L. ATLEE, M. D., Professor of Medical Chemistry in the Med. Dept. of Penn. College, Philadelphia, Pennsylvania.

In an article, contained in the American Journal of Med. Sciences, for April, 1850, Prof. Atlee reports two cases of "large peritoneal section." In one case the section was exploratory; the tumour found to be uterine and fibrous, and not removed. The operation was made 22d May, 1849, the patient recovered from the operation, and died by erysipelas, Nov. 3d, 1849.

In the second case, there existed an unilocular cyst of the the right ovary; weight, forty pounds. Patient recovered.

The length of the reports of these cases, precludes their insertion.

In the same article, Prof. A. presents an analysis of a large number of cases of ovariotory, which is interesting and valuable. The latter we select.

The author embraces the occasion to establish his claim to the fruits of his labors, which, it appears, have been appropriated by Mr. Lee, of London. In justice to Prof. Atlee, we copy that portion of his article.

EDITOR BUFFALO MEDICAL JOURNAL.

"In the *Amer. Journ. of Med. Sciences*, April, 1844, I published a table of 101 cases of Ovariotomy, in which I made a synopsis of the important points of each case. Since the publication of that table, I have been watchfully keeping pace with the operation, and have now tabulated 179 cases. I also made an analysis of that table in order that the profession might see at a glance the most important aspects of this operation prominently arranged. I have done the same with my manuscript table, and will submit it to the profession, in order that they may properly esti mate the present condition of gastrotomy :—

1. Of these 179 cases, 28 were of the minor section, 133 of the major and 18 unknown. Of the minor operation, 20 recovered, and 8 died, or one in every $3\frac{1}{2}$; of the major, 87 recovered, and 46 died, or one in $2\frac{41}{46}$; of the unknown, 13 recovered, and 5 died, or one in $3\frac{3}{5}$. Total, 120 recovered, 59 died, or one in $3\frac{3}{59}$, or 59 in 179 cases, or 32.96 cases in 100.

2. Of the 179 cases, 34 were not completed, or one in $5\frac{9}{34}$; and, in 6, there was no tumour, or one in $29\frac{5}{6}$ cases.

3. Of the 34 unfinished operations, 19 were the large section, 8 the small, and 7 unknown; 14 of the first recovered, 5 died, or one in $3\frac{4}{5}$; 4 of the minor recovered, 4 died, or one in 2; 6 of the unknown recovered, 1 died, or one in 7. Total, 24 recoveries, 10 deaths, or one in $3\frac{2}{5}$ of the unfinished cases.

4. Of the 6 operations in which no tumor was found, 5 were major, and 1 minor; 3 of the former recovered, 2 died; and the minor recovered — making 4 recoveries, 2 deaths, or one in 3 cases.

5. In 17 cases, other important diseases co-existed; in 4 of these, the operation was left unfinished, and all the patients recovered; death occured in all the rest but one. 14 of these cases were the major, 2 the minor, and 1 unknown.

6. In 62 cases there were adhesions; in 41, none; in 76, not stated. Of the first, 36 recovered, 26 died, or one in $2\frac{5}{13}$; of the second, 29 recovered, 12 died, or one in $2\frac{5}{12}$ cases.

7. The cause of death in the 59 fatal cases is recorded as follows : From hemorrhage, 12; peritonitis, 12; exhaustion, 3; shock of operation, 2; inflammation of mucous coat of large intestines, 1; gangrene of peritoneum, 1; peritonitis and gangrene, 1; diarrhœa and peritonitis, 1, peritonitis and constitutional debility, 1; inflammation of lungs, 1; ileus and phlebitis of lower limbs, 1; a fall during convalescenee, 1; causes not stated, 21. Total, 59.

8. The period of death after the operation in 59 fatal cases is recorded as follows : Died the 70th day, 1; in 6 weeks, 2; in 3 weeks, 1; the 17th day, 1; the 15th day, 1; the 14th day, 1; the 10th day, 1; the 9th day, 1; the 7th day, 3; the 6th day, 5; the 5th day, 2; in 3 days, 3; in 74 hours, 1; in two days, 1; in 44 hours, 1; in 36 hours, 5; in 32 hours, 1; in 30 hours, 1; in 17 hours, 1; in 12 hours, 2; in 11 hours, 1; in 8 hours 1; in 6 hours, 1; in 4 hours, 1; immediately, 2; time not stated, 18 Total, 59. The average time of death in 41 cases stated, 8 days.

9. Of the 17 cases complicated with other important diseases, 7 were manifestly not proper for the operation, and 8 others, instead of 4, ought to have remained unfinished after the abdominal section was made. Throwing the first 7 cases out of the estimate, would leave 172 legitimate cases, and rating the 4 others, that ought to have remained unfiinished, according to the mortality of unfinished operations, it would make 123 recoveries, and 49 deaths, or one in $3\frac{25}{49}$, or $28\frac{21}{43}$ deaths in 100 cases, which I consider the correct rate of mortality of the operation as it is represented by my manuscript table.

10. Under the head of the 8th paragraph, I have stated that death occurred, in one instance, on the 70th day; in two instances, after the expiration of 6 weeks, and, in another case, from a fall during convalescence. Now, I would ask, is it proper to consider the fatal termination in these cases the result of the operation ? Or rather, ought they not to be considered as having recovered from the *operation*, and be so reported? If so, then the fairest estimate would be, after throwing out the 7 cases referred to, 127 recoveries, and 45 deaths; or one in $3\frac{37}{45}$, or $26\frac{7}{13}$ deaths in 100 cases.

11. The rate of mortality has been very much diminished since the publication of my table in 1845. Then, there was one death in every $2\frac{25}{38}$ cases of gastrotomy, or 37.62 deaths in every 100 cases. Since the publication of that table, 78 cases have occurred, in which there was one death in every $3\frac{5}{7}$ cases, or 26.92 deaths in every 100 cases. A diminution of nearly 40 per cent. in the rate of mortality.

12. There has also been a diminution in the proportion of unfinished operations, and, in no case since, has the abdomen been opened for the purpose of removing a tumour when no tumour could be found. It should also be observed that several of the more recent unfinished operations have been of the exploratory character. Hence, diagnosis has also improved.

13. If we compare the above statistics of the mortality of the operation for ovariotomy, with those of the mortality following other capital operations, we will find them in favor of gastrotomy.

Take, for instance, the valuable tables of cases of operation on the larger arteries, published by George W. Norris, M. D., in the *Amer. Journ. of Med. Sciences*. The rate of mortality in these cases is, one in $2\frac{95}{96}$ or 33.45 deaths in 100 cases, while, in all the cases of ovariotomy reported, it is one in $3\frac{2}{69}$, or ,32.96 cases in 100. Taking, however, the more recent cases, as noticed in paragraph 11, the rate of mortality is 25 per cent. greater in the operation on the larger arteries than in gastrotomy.

Dr. Lee, in his work on "Tumours on the Uterus," refers to some statistics by Malgaigne on Amputations, as published in the *Medical Gazette*, 1846. These amputations were performed in the Parisian hospitals from 1836 to 1841, and were 852 in number. They included *all* the extremities, even to fingers and toes. Of these, 332 died, or one in $2\frac{47}{83}$, or 38.97 deaths in every 100 cases. A mortality nearly 45 per cent. greater than that of gastrotomy.

201 of these amputations were of the thigh. Of these, 126 died, or one in $I\frac{25}{42}$, or 62.68 deaths in every 100 cases. Exceeding the mortality of gastrotomy 133 per cent.

192 amputations were of the leg; 106 died, or one in $1\frac{43}{53}$, or 55.21 deaths in every 100 cases; 82 per cent. greater than gastrotomy.

Before concluding tnis paper, I trust it will not be thought improper, in connection with the above statistics, to allude to a matter of a personal character. In the *Amer. Journ. of Med. Sciences* for April, 1845, page 330, I published a table of cases of ovariotomy that had occurred up to that date, the construction of which, cost much trouble, time, and labor. This table was prepared, at first, solely for my own use, for the convenience of reference; but, believing it would be of service to the profession in the discussion of the question of ovariotomy, I offered it for publication. In January, 1847, "*A Dissertation upon Tumours of the Uterus and its Appendages*" was published by Mr. Thomas Safford Lee, London, for which the Jacksonian Prize was awarded. It is a valuable publication, the most complete epitome of knowledge on ovarian dropsy we possess, and enters into the question of gastrotomy more fully than any treatise of the kind. At page 183, Mr. Lee says, "*I have carefully collected into a tabular form all the known operations for the extraction of the ovary*," and then, with the addition of a few cases which occurred since April, 1845, he gives, as the result of his own labor, the very table which I had published in 1845, omiting any acknowledgement whatever. After M . Lee enters upon the discussion of the question, on page 183, "*What are the results of the operation already performed?*" he makes use, very frequently, of the facts which I had collected, adopting my arrangement of them, and even my language, without any reference to the source whence he mainly drew the information, upon which were founded the valuable deductions in his book. Feeling aggrieved that my labors had been appropriated without the credit, to which I was justly entitled, having been awarded, I addressed a letter to Mr. Lee, appealing to his sense of justice, and calling his attention to the *omission*. Mr. Lee promptly replied, assuring me of his regret that such an omission should have occurred, and sincerely hoped that I would not consider it a wilful one, acknowledging his indebtedness to me, and promising, should occasion offer, in a second edition of this work, to do me ample justice for this temporary omission. From the gentlemanlike character of Mr. Lee's letter, I was satisfied that the omission was not intentional, and felt willing that the correction be made by himself at the proper time, and so had determined. I think it necessary, however, in justice to myself, to refer to this subject at this time, inasmuch as a distinguished American author has given to Mr. Lee the credit of presenting those very facts to the profession. In a work entitled "*Females and their Diseases, a series of Letters to his Class*," by Charles D. Meigs, M. D., &c. &c., Philadelphia, 1848, page 315, are these words: "Facts are the things that teach—and I shall close this letter by laying before you the tabular view presented by Dr. T. S. Lee, who, I am sure, will not object to my using so great liberty with his work, the more especially as it may assist in spreading further and wider the knowledge he has been at so great pains to collect, and make it both more public and useful at once." Then follows *my* table with this head, "*Table, by Dr. Lee*," and the several succeeding pages, through which the table is continued, have, each one, this title : "*Dr. Lee's Table.*" As both Dr. Meigs' book and the *Amer. Med. Journ.* have a wide circulation among American practitioners, it will be perfectly competent for them to compare Dr. Lee's table of 1847 with my table of 1845, and decide *who* "*presented* the Tabular View," and *who* "has been at *so great pains to collect* the knowledge" therein contained."

